# Supplementary material for: Poly I:C Pre-Treatment Induced the Anti-Viral Interferon Response in Airway Epithelial Cells
Source: Viruses. 2023 Nov 27;15(12):2328. doi: 10.3390/v15122328 (PMC10747011; doi:10.3390/v15122328)
Supplement: Supplementary file 1 [file viruses-15-02328-s001.zip › viruses-2692373-supplementary.pdf]

Table S1. Single data on asthma and control patients.

Patient ID	Gender	Age (years)	BMI (kg/m²)	Airway infection in the last year	Allergic rhinitis	Asthma medication	Family history of atopy/ asthma	FEV1 (%)	FVC (%)	FEV1/FVC (%)	Phenotype (PRACTALL 2008)
A1	male	28	44,79	2	yes	ICS+LABA	yes	95	89	87,58	mixed
A2	female	33	24,68	1	yes	ICS+LABA	no	126	120	86,81	allergic
A3	male	63	29,3	0	yes	ICS+LABA+ Reproterol+ Cromoglycin	yes	89	98	69,17	allergic
A4	male	53	25,31	0	yes	ICS+LABA	yes	80	90	70,34	mixed
A5	male	57	33,21	0	no	ICS+LABA	yes	76	91	64,24	mixed
A6	female	47	29,07	3	no	ICS+LABA	yes	113	102	88,09	mixed
A7	female	40	25,7	3	no	ICS+LABA	no	84	89	77,67	mixed
A8	male	60	25,6	0	yes	no	yes	99	109	69,79	allergic
A9	female	44	24,5	0	no	ICS+LABA	no	82	81	81,84	mixed
A10	female	51	27,9	1	yes	ICS+LABA	no	91	96	75,51	allergic
A11	male	26	25,8	4	no	no	yes	74	90	65,23	allergic
A12	female	56	25,7	1	yes	no	no	86	86	79,15	mixed
A13	male	31	21,8	2	yes	ICS+LABA	yes	100	101	81,65	mixed
A14	male	25	22,79	0	yes	ICS	yes	97	86	94,54	allergic
A15	female	47	25,62	2	yes	ICS+LABA	yes	89	85	82,79	allergic
A16	female	26	22,46	6	yes	no	no	96	105	77,94	mixed
A17	female	54	21,97	0	yes	ICS+LABA	no	96	98	77,25	allergic
A18	female	47	26,18	0	yes	ICS+LABA	no	117	119	78,82	allergic
A19	female	24	23,03	1	yes	ICS+LABA	no	104	105	84,6	mixed
A20	male	23	29,18	1	yes	no	yes	91	101	75,16	allergic
A21	male	41	30,15	0	yes	ICS+LABA	no	77	84	72,05	allergic
A22	female	52	23,8	3	yes	ICS+LABA	yes	104	94	87,85	allergic
C1	female	34	23,05	0	no	no	yes	96	90	88,87	/
C2	female	33	20,58	0	no	no	no	111	105	88	/
C3	female	29	18,26	1	no	no	no	84	80	89,12	/
C4	female	44	21,95	0	no	no	no	104	104	80,18	/
C5	male	62	21	2	no	no	no	96	96	76,98	/
C6	male	64	29,3	1	no	no	no	126	116	82,84	/
C7	male	26	27,7	1	no	no	no	87	94	76,12	/
C8	female	23	21,5	2	no	no	yes	90	99	77,64	/
C9	female	63	18,6	2	no	no	yes	99	83	93,96	/
C10	female	53	24,8	0	no	no	no	110	106	82,96	/
C11	male	55	28,4	0	no	no	no	96	86	85,71	/
C12	male	21	23,5	0	no	no	yes	100	107	77,58	/
C13	female	22	20,4	2	no	no	no	102	91	97,24	/
C14	male	31	32,5	0	no	no	no	111	104	87,65	/
C15	male	25	21,1	0	no	no	no	93	106	71,81	/
C16	male	43	28,4	0	no	no	no	116	111	84,06	/
C17	female	28	17,15	0	no	no	no	86	102	71,85	/
C18	female	61	22,32	0	no	no	no	106	97	85,49	/
C19	male	24	26,44	2	no	no	no	98	117	69,38	/

Table S2. Cell culture reagents used in this study.

Product	Company	Catalog number
RPMI 1640 Medium, no glucose	Gibco™, Thermo Fisher Scientific	11879020
UTM tubes	Copan	330C
DNAase I	Sigma-Aldrich	10104159001
Collagen R 2mg/ml	Serva	47254
48 Well Cell Culture Multiwell Plate, CELLSTAR®, with Lid	Greiner Bio-one	677180
PneumoCult™-Ex-Plus Medium	Stemcell™ Technologies	05040
Antibiotic-Antimycotic (100X)	Gibco™ Thermo Fisher Scientific	11570486
Gentamicin (10mg/ml)	Sigma-Aldrich	G1272
FBS Superior	Sigma-Aldrich	S0615
Sodium Bicarbonat 7,5%	Gibco™ Thermo Fisher Scientific	25080060
6 Well Cell Culture Multiwell Plate, CELLSTAR®, with Lid	Greiner Bio-one	657160
RPMI 1640 Medium	Gibco™, Thermo Fisher Scientific	21875091
RPMI 1640 Medium, no glucose	Gibco™, Thermo Fisher Scientific	11879020
Penicillin-Streptomycin Solution 100X	Anprotec	AC-AB-0024
L-Glutamine 100X, 200mM	Anprotec	AC-AS-0001
Poly I:C (polyinosinic-polycytidylic acid potassium salt)	Sigma-Aldrich	P9582-50MG
Trypsin-EDTA 1X in PBS w/o Calcium w/o Magnesium w/o Phenol Red	Anprotec	AC-EZ-0009
Trypan Blue Solution	Sigma-Aldrich	T8154
iTaq™ Universal SYBR® Green Supermix	Bio-Rad Laboratories	1725124
QIAzol Lysis® Reagent	Qiagen (Maryland, USA)	79306
RevertAid™ First Strand cDNA Synthesis Kit	Thermo Scientific™	K1622
human IL-29/IL-28B (IFN-lambda 1/3) DuoSet ELISA	R&D Systems	DY1598B
PBS (Dulbecco's Phosphate Buffered Saline w/o Calcium w/o Magnesium)	Anprotec	AC-BS-0002

Table S3. Human qPCR primers.

Gene	Primer	Sequence (5′ -> 3′)
HPRT	fwd	TGA CAC TGG CAA AAC AAT GCA
	rev	GGT CCT TTT CAC CAG CAA GCT
IFN-α	fwd	AGG AGT TTG ATG GCA ACC AG
	rev	CTC TCC TCC TGC ATC ACA CA
IFN-β	fwd	AGT AGG CGA CAC TGT TCG TG
	rev	AGC CTC CCA TTC AAT TGC CA
IFN-λ	fwd	CTG CCA CAT AGC CCA GTT CA
	rev	AGC GAC TCT TCT AAG GCA TCT
IFNαRII	fwd	ATG CTT TTG AGC CAG AAT GCC
	rev	CAT CTG TGT AAT CAG GCG AAT CA
OAS-1	fwd	AGC TGG AAG CCT GTC AAA GA
	rev	GGT TTA TAG CCG CCA GTC AA
TLR3	fwd	GTA TTG CCT GGT TTG TTA ATT GG
	rev	AAG AGT TCA AAG GGG GCA CT
